# Correlates of non-institutional delivery to delayed initiation of breastfeeding in Nigeria: logit-decomposition and subnational analysis of population-based survey

**DOI:** 10.1186/s41043-023-00466-3

**Published:** 2023-11-06

**Authors:** Oyewole K. Oyedele

**Affiliations:** 1 International Research Centre of Excellence, Institute of Human Virology, Nigeria (IHVN), Abuja, FCT Nigeria; 2 Department of Epidemiology and Medical Statistics, Faculty of Public Health, College of Medicine, University of Ibadan, Ibadan, Nigeria

**Keywords:** Breastfeeding, Home birth, Facility delivery, Caesarian section, Skin-to-skin contact, Logistic regression, Multivariate decomposition

## Abstract

**Background:**

Studies have connected newborn delivery settings and modality to optimal breastfeeding, but how it influences untimely initiation, mostly prevalent in sub-Saharan Africa is unknown. Hence, the role of home delivery on delay initiation of breastfeeding (DIBF) in Nigeria was investigated to inform evidence-based strategy for improved breastfeeding practice.

**Methodology:**

This is a secondary analysis of births (11,469 home and 7632 facility delivery) by 19,101 reproductive age women in the 2018 NDHS. DIBF is the outcome, home birth is the exposure, and explanatory variables were classified as: socio-demographics, obstetrics and economic factors. Descriptive statistics (frequencies and percentages) were reported, and bivariate (chi-square) analysis was carried out at 20% (*p* < 0.20) cutoff point. Multivariable logistic regression assessed the probability and significance of the outcome per place of birth. Multivariate decomposition further evaluated the endowment and coefficient effect contribution by independent factors to the outcome. Analysis was carried out at *p* < 0.05 (95% confidence level) on Stata.

**Results:**

56.6% of mothers DIBF, with 37.1% and 19.5% from home and facility delivery, respectively. Home delivery (AOR = 1.34, 95% CI 1.17–1.52) increase the chance of DIBF by 34%, while DIBF probability reduces by 26% in facility delivery (AOR = 0.74, 95% CI 0.65–0.85). DIBF is 5 times more likely in caesarian section delivery (AOR = 5.10, 95% CI 4.08–6.38) compared to virginal birth in facility delivery. Skilled antenatal provider, parity and wealth are negatively associated with DIBF in home birth, while undesired pregnancy, rural residency, partial/no skin-to-skin contact and large child size positively influence DIBF in both home and facility delivery. Skilled antenatal provider (*C* =  − 66.3%, *p* < 0.01) and skin-to-skin contact (*C* =  − 60.6%, *p* < 0.001) contributed most to reducing the negative DIBF effect with 69% and 31% overall characteristics and coefficient effect component, respectively. DIBF is more likely in Bauchi and Sokoto but less likely in Bayelsa.

**Conclusions:**

High DIBF prevalent in Nigeria was largely due to elevated rate of home birth, positively associated with DIBF. Caesarian section delivery though heightens the chance of DIBF in facility delivery. Strengthening utilization of skilled provider and skin-to-skin contact can eliminate two-third of the adverse DIBF effect and improve early initiation rate. Adopting this strategy will bridge home-facility delivery gap to achieve optimal breastfeeding practice.

**Supplementary Information:**

The online version contains supplementary material available at 10.1186/s41043-023-00466-3.

## Introduction

Timely initiation of breastfeeding (TIBF) within the first hour of life was recommended by WHO and UNICEF to provide child with required immunity against disease and consequently lower the risk of neonatal and post-neonatal death [1–5]. Mother also benefits from the breastfeeding practice through involution of the uterus, reduced risk of: postpartum hemorrhage, high blood pressure and depression and to facilitate mother and child bonding while prolonging breastfeeding duration to influence child spacing and reduce the risk of ovarian and breast cancer [6, 7].

Despite these enormous health benefits, only 50% of newborn are put to breast within the first hour of life globally and the prevalence is even lower in sub Saharan Africa (SSA) [8]. TIBF prevalence in 2010–2015 varied between 37.8% (24.6–51.1) in Central Africa and 69.3% (67.6–70.9) in Southern Africa, and a pooled prevalence of 58.3% (58.0–58.6) was recently reported in SSA countries [9, 10]. Studies opined that optimal breastfeeding practice can prevent over 800,000 neonates death due to the delayed initiation of breastfeeding (DIBF) [10, 11]. The prevailing DIBF, particularly in SSA, is a threat to increased risk of neonatal morbidity and mortality by increase in the risk of infection and therefore decrease the chance of infant survival [5, 12].

In Nigeria, the prevalence of TIBF has though increased by 9% from 33% in 2013 to 42% in 2018, but prelacteal feeding only reduced from 56 to 49% in the last decade [13, 14]. Also, facility-based delivery has slightly increased from 36% in 2013 to 39% in 2018, while neonatal (NMR), infant (IMR) and under-5 mortality rate (U5MR) are currently: 39/1000, 67/1000 and 132/1000 livebirths, respectively [14]. However, these statistics are below the WHO expected 50% coverage of TIBF by 2025 and such little increase over a long time will dampen the sustainable development goal (SDG) targeted toward reducing neonatal, infant and under-5 mortality by 2030 [15, 16].

Studies have documented that economic, maternal and health-related factors are associated with early breastfeeding initiation in Nigeria and SSA [3, 8, 10, 17–24]. These include: health facility delivery [3, 8, 10, 17–20], vaginal delivery [8, 10, 18, 19, 21], use of skilled attendants at childbirth [21, 23, 25], parity [10, 18, 19, 21, 23], singleton birth [3, 8, 10], child size at birth [10, 18, 21], antenatal care visit [3, 10, 21, 22], household wealth [10, 18, 21, 23], residence [3, 18–22], education [3, 10, 24]. Also, the importance of the skin-to-skin contact on early breastfeeding initiation in Nigeria has been emphasized [26–28]. However, studies generally connected type of deliveries, mode of births and socio-economic inequalities to the practice of breastfeeding and prelacteal feeding [2, 17, 29–31]. However, there is limited evidence on its influence on delayed breastfeeding initiation.

Furthermore, studies independently thrive in examining home and facility delivery including utilization of skilled delivery in Nigeria [32–38]. Nevertheless, the clustered confounding impact of the place of newborn delivery on breastfeeding initiation is yet to be determined particularly, with Nigeria among the top five countries with the highest burden of neonatal deaths worldwide [16]. Hence, there is the need to examine the facility and non-facility delivery gap contribution to DIBF. Also, the subnational distribution of DIBF in Nigeria is unknown. Thus, this study decomposed the effect of DIBF by place of delivery of newborns and provides the subnational prevalence to respond to the following research questions; is there any difference in the prevalence of DIBF between home and health facility births in Nigeria? What is the effect of non-institutional delivery on the delayed breastfeeding initiation for newborns? What are the factors contributing to the non-institutional delivery effect on DIBF? The study findings will inform interventional strategic support to alleviate breastfeeding inequity through program action that will improve practice.

## Method

### Study design, data source and settings

This is a secondary analysis of the cross-sectional survey data extracted from the nationally representative Nigerian Demographic and Health Survey (NDHS). The NDHS data have been collected in a five-year interval since 2003 after the first episode in 1990, with the 2018 NDHS being the most recent. Nigeria comprises of 36 states and the federal capital territory hosted in six geopolitical zones: northeast, northwest, northcentral, southeast, southwest and south–south. The country population being the largest in Africa is presently the inhabitants of over 200 million people, and it is projected to double along with other African countries by 2050 [39].

### Sampling strategy and participants

**Fig. 1:**
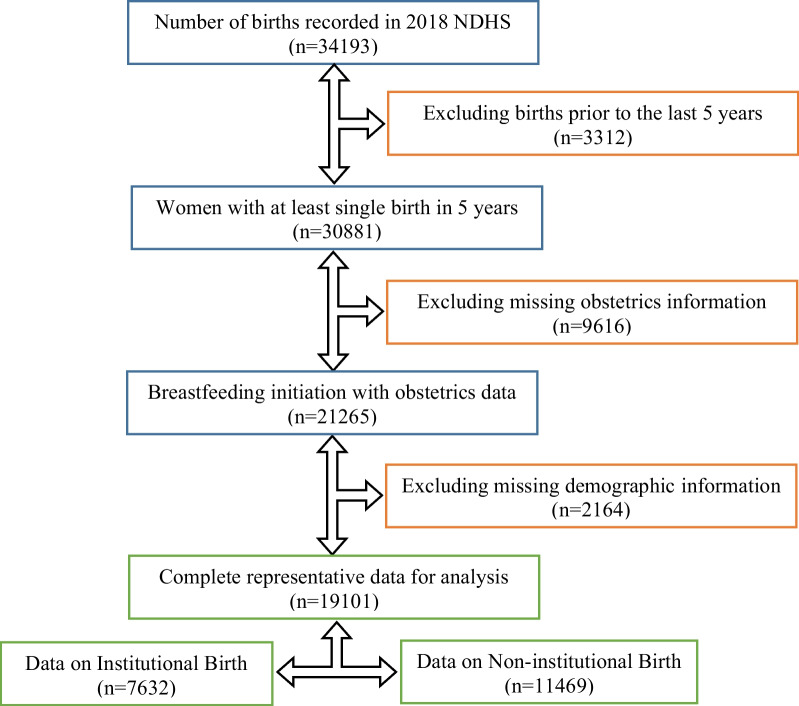
Data flow schema for the sample data excluded and included in the study analysis (NDHS 2018)

The two-stage stratified random sampling technique was the sampling strategy for data collection in the 2018 NDHS. This was based on the sampling frame from the National Population and Housing Census (NPHC). Administratively, Nigeria states are divided into local government areas in the first sampling stage that contains 74 selected strata. The second stage was the subdivision of the administrative units into rural and urban enumeration areas consisting of 1400 urban and rural clusters referred to as the primary sampling units. Households were selected per cluster by equal probability systematic sampling, and a total of 42,000 households were selected in which  about 48,000 women of reproductive age (15–49 years) who had at least a birth in the last five-years preceding the survey were interviewed via questionnaire administration on infant and young child feeding (IYCF) practice, including breastfeeding initiation as well as the underlying maternal and child factors. Hence, 19,101 (7632 in facility and 11,469 in home birth) completed responses were analyzed in the study (Fig. 1). The 2018 NDHS achieved a response rate of about 99% as documented along with the details about the survey sampling methodology [13, 14].

### Outcome variable

Outcome variable is the ‘delayed initiation of breastfeeding (DIBF)’ which was measured from the response to the question; how long after birth do you first introduce breastmilk for child? This was classified according to the WHO and UNICEF standards as: ‘timely’ if it is within the first hour of birth and ‘delayed’ if initiation was after the first hour of birth as illustrated below (1).

$${\text{TIBF}} = \left\{ {\begin{array}{*{20}c} {0,\quad {\text{Timely}}\quad {\text{i}}.{\text{e}}{.}\;{\text{within}}\;{\text{the}}\;{\text{first}}\;{\text{hour}}\;{\text{of}}\;{\text{birth}}} \\ {1,\quad {\text{delayed}}\quad {\text{i}}.{\text{e}}{.}\;{\text{after}}\;{\text{the}}\;{\text{first}}\;{\text{hour}}\;{\text{of}}\;{\text{birth}}} \\ \end{array} } \right.$$

### Exposure variable

Place of delivery (non-institutional, institutional) [3, 8, 10, 18] is the exposure variable, and women who had the last singleton or multiple birth at home or in a non-hospital settings are the exposed, while those who had the last childbirth at a healthcare facility are the unexposed.

### Explanatory variables

Independent variables were selected based on the factors that were measured in previous studies investigating delayed initiation of breastfeeding [11, 12, 17, 29]. These were defined under the domains or categories of respondents’ demographic, community characteristics, obstetrics and economic-related factors as highlighted below.

#### Demographics and community characteristics

Age group in years [15–49]; place of residence (urban, rural); education (no formal education, primary, secondary, tertiary); marital status (married, unmarried); partner education (no formal education, primary, secondary, tertiary); religion (Christianity, Islam, traditional/other); ethnicity (house/Fulani, Igbo, Yoruba); and region (northcentral, northeast, northwest, southeast, south–south, southwest).

#### Obstetrics and reproductive health factors

Pregnancy desire (then, later, no more); ANC visit (none, < 4 visit, 4+ visit); prenatal provider (unskilled, skilled), SBA use (no, yes); parity (primiparous, multiparous); Delivery by CS (no, yes); birth type (single birth, twin/multiple births); sex of child (male, female); child size (small, average, large); and skin-to-skin contact (put to chest touching bare skin, put to chest not touching bare skin, not put to chest).

#### Economic-related characteristics

Occupation (unemployed, employed); wealth (poor, average, rich); and media exposure (no, yes).

### Statistical analysis

The descriptive analysis was primarily performed to compute the frequency and percentage of women by characteristics and viz-a-viz their exposure status (home or facility delivery). The mean (± standard deviation) summarized the numerical variable. Breastfeeding initiation was classified as timely (with code 0) and delayed (with code 1), which was summarized in proportion. Also, summary statistics of the exposure group (proportion of home births) and the unexposed group (proportion of facility-based delivery) was reported.

Bivariate analysis was then conducted to initially assess the association between women DIBF status and their maternal characteristics. This was done for each of the type of place of delivery (home and facility) based on the statistics of the observed and expected count and variables were considered important at a 20% cutoff point (*p* < 0.20). Factors identified to be associated with the outcome at this cutoff point were included in the succeeding multivariable analysis. Pearson chi-square statistics were reported throughout as none of the 20% expected cell count was less than 5.

Odds measuring the association between DIBF and maternal factors identified in the bivariate analysis were assessed in the multivariable logistic regression. Both the adjusted and crude odds ratio were reported to quantify the likelihood and significant (*p* < 0.05) of the predictors when other independent variables were controlled or uncontrolled in the model, respectively. Similarly, this analysis was performed for the disaggregated place of delivery. Delivery via caesarian section was not applicable and thus omitted in the non-institutional group analysis. Insignificant variables (media exposure and child sex) were not included in the analysis of the institutional delivery group, while birth type was not included in the group analysis of home and facility delivery.

Decomposition analysis specifically multivariate decomposition analysis (MDA) was performed to evaluate the factors contributing to non-institutional delivery (exposure) effect on the delayed breastfeeding initiation (outcome). Both the endowment and coefficient effect component were reported based on the percentage (%) contribution and significant (*p* < 0.05). The confounding effect of CS delivery was isolated in the decomposition analysis as it is associated with both the exposure and the outcome. The data were weighted using the women weighting factor in the DHS to correct for heterogeneous sample due to the complexity of the survey design. The svy command was used during the analysis to adjust for the sample weight, strata and cluster. All analysis was performed using Stata (version 17.0) at a 95% confidence level (5% level of significance).

### Multivariable regression

Multiple logistic regression model was applied to model the binary response [*P*($Y_{i} = 0\;{\text{if}}\;{\text{early}}$), *P*($Y_{i} = 1\;{\text{if}}\;{\text{late}}$)] in which the estimate of the regression coefficients due to the shape parameter (logistic curve) can be obtained under the maximum likelihood estimator compared to the least square estimator in the linear regression. The multivariable logistic regression model equation is illustrated below as the linear combination of the regression coefficients ‘*β*’ and predictors ‘*X*.’

1 $${Y}_{i}=\mathrm{ ln}\left(\frac{p}{1-p}\right)= {\beta }_{0}+ {\beta }_{1}{X}_{1i}+\dots + {\beta }_{p}{X}_{pi}+ \varepsilon$$

2 $$E\left( {Y_{i} } \right) = p_{i} = \frac{{\exp \left( {\beta_{0} + \beta_{1} x_{pi} + \cdots + \beta_{p} x_{pi} } \right)}}{{1 + \exp \left( {\beta_{0} + \beta_{1} x_{1i} + \cdots + \beta_{p} x_{pi} } \right)}}$$

where $\mathrm{ln}\left(\frac{p}{1-p}\right)$ is the log odds (*p* is the probability of success (i.e., delayed initiation of breastfeeding) and 1 − *p* is the failure probability (i.e., timely initiation of breastfeeding)). ${\beta }_{0}$ is the logistic regression constant or intercept. ${\beta }_{1}+\dots + {\beta }_{p}$ are the *p* × 1 vector of regression coefficient or slopes. ${X}_{i1}+\dots +{X}_{ip}$ are the *nxp* matrix of explanatory variables predicting the log odds in the model.

### Decomposition analysis

The multivariate decomposition analysis procedure determines the component effect and further partitions the component into endowment and coefficients effect was adopted [40]. MDA extended to decompose nonlinear logit and probit models to analyze the variable composition and effect attributed to the group difference or trends spanning over time to explain the root factors [36, 38, 40, 41]. This method was applied in this study to decompose the logit model of the binary outcome group (delayed breastfeeding initiation in reference to the early breastfeeding initiation). The group (institutional and non-institutional) decomposition of the logit model is represented in the set of equations below.

3 $$Y = F\left( {X\beta } \right)$$

4 $$Y_{P} - Y_{1 - P} = F\left( {X_{P} \beta_{P} } \right) - F\left( {X_{1 - P} \beta_{1 - P} } \right)$$

5 $$Y_{P} - Y_{1 - P} \equiv\, \left\{ {F\left( {X_{P} \beta_{P} } \right) - F\left( {X_{1 - P} \beta_{P} } \right)} \right\} + \left\{ {F\left( {X_{1 - P} \beta_{P} } \right) - F\left( {X_{1 - P} \beta_{1 - P} } \right)} \right\}$$

where *Y* is the *n* × 1 vector of the dependent variable 0 ≤ *p* ≤ 1, *X* is the *n* × p matrices of the independent variables, and *β* is the *p* × 1 vector of the regression coefficients in (1). The difference in the DIBF proportion was decomposed by home or hospital birth in (2), and in (3) the component {*F* ($X_{P} \beta_{P}$) – *F* ($X_{1 - P} \beta_{P}$)} refers to the differential attributable to the endowment component (explained composition), while {*F* ($X_{1 - P} \beta_{P}$) –* F* ($X_{1 - P} \beta_{1 - P}$)} refers to the differential attributable to the coefficients component (unexplained composition). $Y_{P}$ denotes the proportion of DIBF (high-outcome group), while $Y_{1 - p}$ denotes the proportion of TIBF (comparison group).

## Result

### Institutional versus non-institutional delivery

**Fig. 2:**
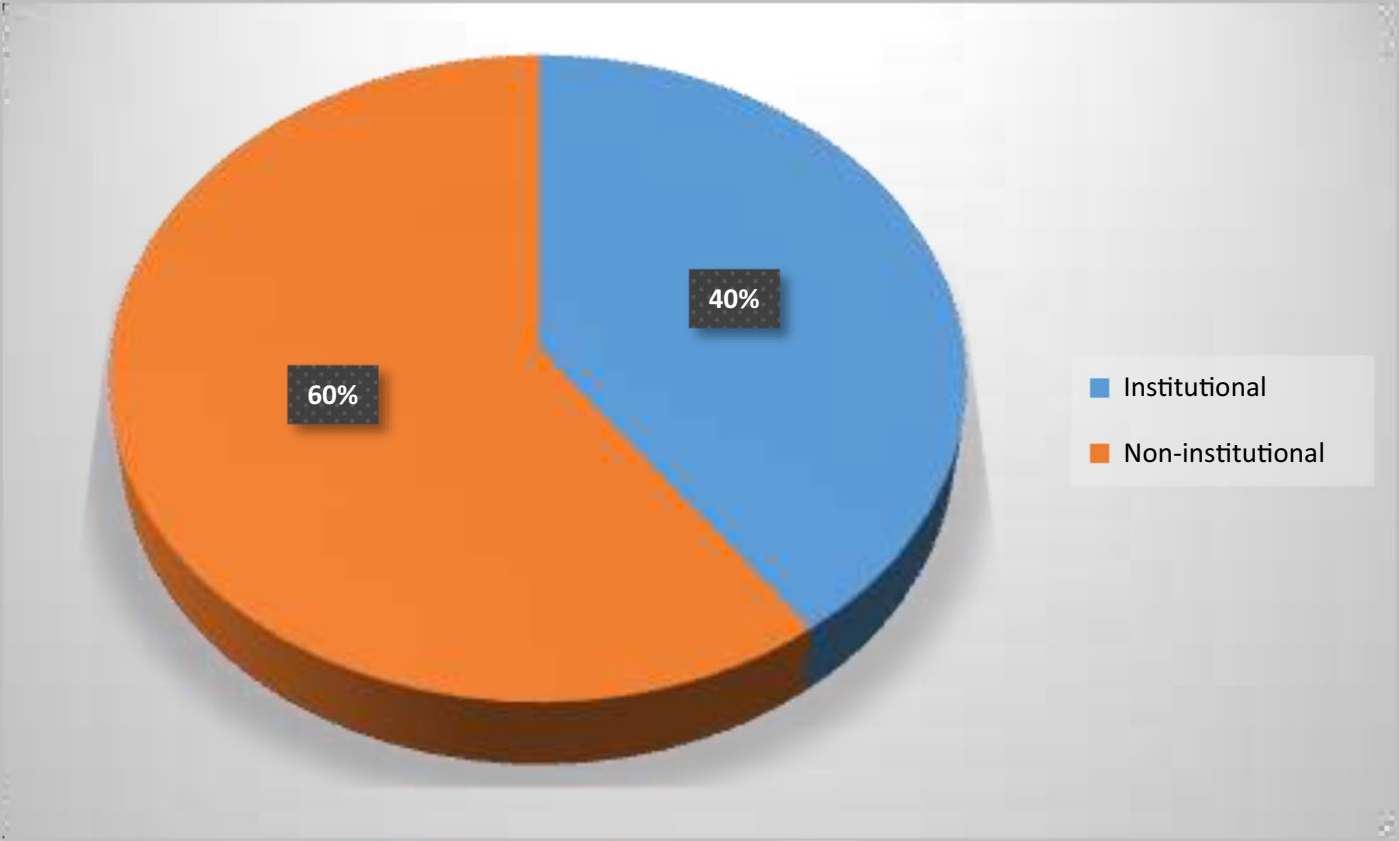
Distribution of the type of place of delivery in Nigeria (Data from the 2018 NDHS)

Figure 2 shows the proportion of women who had hospital-based delivery versus those who had home or traditional birth. About 60% (11,469) of deliveries occur in non-institutional settings compared to the 40% (7632) institutional or hospital deliveries.

### Prevalence of DIBF by place of delivery

**Fig. 3:**
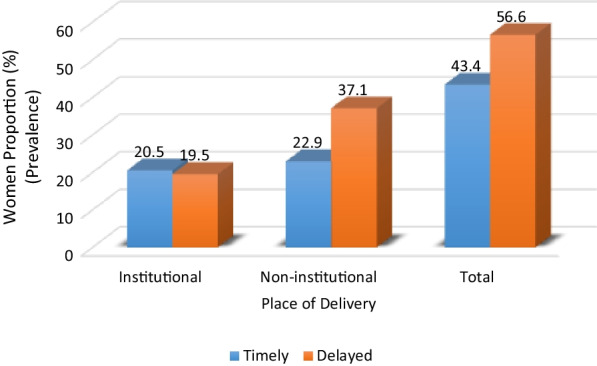
Prevalence of breastfeeding initiation by place of delivery among women with at least single birth prior to the 2018 NDHS

Figure 3 reveals the prevalence of DIBF in institutional and non-institutional deliveries. Of the 40% (7632) that had institutional delivery, less than half (19.5%) delayed breastfeeding initiation, whereas breastfeeding initiation was delayed by about two-third (37.1%) of the 60% (11,469) that had home or traditional births.

### Subnational prevalence of DIBF by place of delivery

**Fig. 4:**
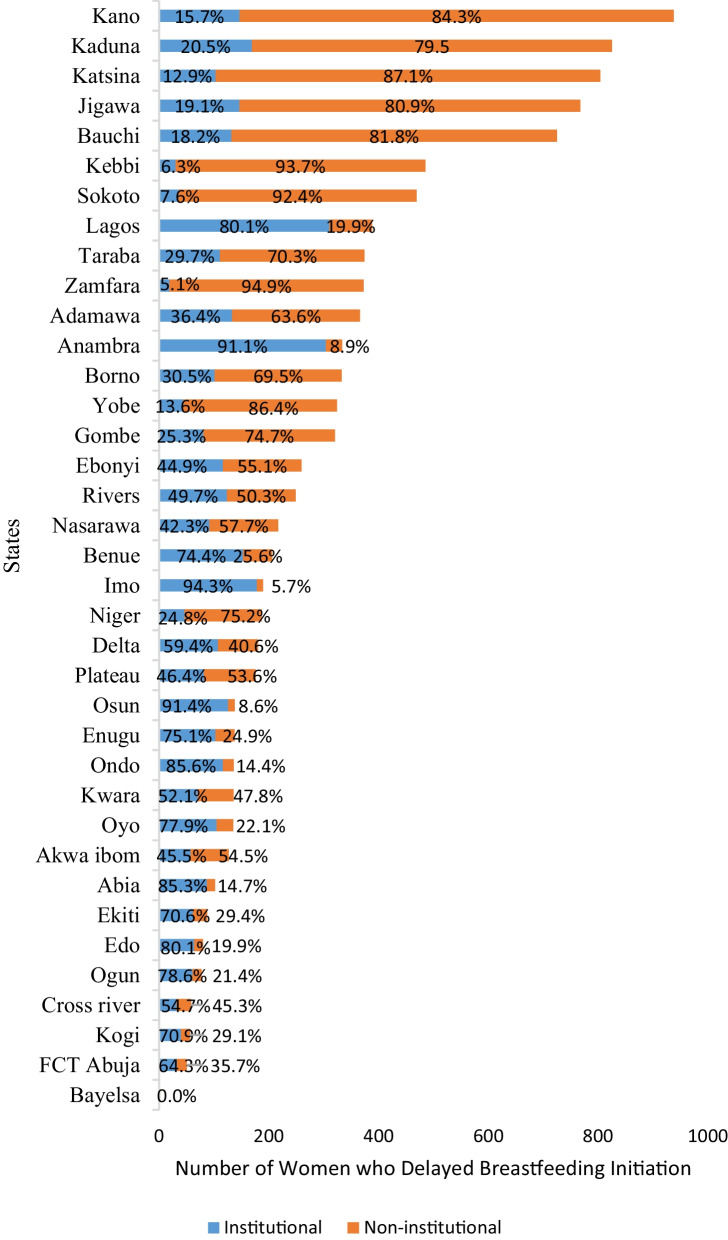
Subnational prevalence of delayed initiation of breastfeeding by place of delivery among women of childbearing age (NDHS 2018)

State-level prevalence of DIBF by delivery place is shown in Fig. 4. DIBF is highest in Zamfara (94.9% from home birth and 5.1% from hospital delivery), followed by Kebbi (93.7% and 6.3% from home and hospital birth, respectively) and Katsina and Kano with 87.1% and 84.3% home birth prevalence, respectively. DIBF was lowest (0% in home birth) in Bayelsa state.

### Descriptive statistics by place of delivery

Table 1 shows the descriptive statistics of maternal characteristics by place of delivery. Overall, 60% (11,469) and 40% (7632) had home and hospital deliveries, respectively. Highest proportion (48.5%) of age-group are women 25–34 years. About 61.3% of the women lives in the rural (45.3% had home birth) while 38.7% of the women resides in the urban (23.9% had hospital birth) (Table 1). Only 8.7% (7.7% had hospital birth) had tertiary education, while 36.9% (32.3% had home birth) had no formal education. Around 97% (18,528) are married, while only 3% (573) are not married. Nearly 45% (about 38% had home delivery) are poor, 20% are average and not less than 35% (up to 25% had hospital birth) are rich (Table 1). About 24.5% (23.4% had home births) had no ANC visit while 57.6% (about 40% had hospital birth) had 4 or more ANC visits. Around 35.3% (31.8% in home delivery) and 64.7% (12,352) had prenatal care from unskilled and skilled provider, respectively (Table 1). SBA use at birth was 41.4% (37.4% in hospital delivery) and about 84.5% (16,141) of the women have had 2 or more births. All 3.1% (593) that had CS delivery are hospital birth. Around 85.3% (16,294) put child to chest and touching bare skin, and 2.8% (1.7% in hospital delivery and 1.1% in home birth) put child to chest but not touching bare skin, while newborns of 11.9% (2273) of the women were not put to chest after birth (Table 1).

**Table 1:** Descriptive statistics by place of delivery

Factors	Non-institutional deliveries *n* (%)	Institutional deliveries *n* (%)	All deliveries *n* (%)
*Age group*			
15–24	3110 (16.3)	1459 (7.6)	4568 (23.9)
25–34	5286 (27.6)	377 (20.8)	9263 (48.5)
35–49	3073 (16.1)	2196 (11.5)	5270 (27.6)
*Place of residence*			
Urban	2824 (14.7)	4562 (23.9)	7386 (38.7)
Rural	8645 (45.3)	3070 (16.1)	11,715 (61.3)
*Education*			
No formal education	7585 (39.7)	1285 (6.7)	8870 (46.4)
Primary	1649 (8.6)	1129 (5.9)	2778 (14.6)
Secondary	2036 (10.7)	3752 (19.6)	5788 (30.3)
Tertiary	199 (1.0)	1465 (7.7)	1664 (8.7)
*Marital status*			
Married	11,217 (58.7)	7311 (38.3)	18,528 (97.0)
Unmarried	252 (1.3)	321 (1.7)	573 (3.0)
*Partner education*			
No formal education	6181 (32.3)	870 (4.5)	7051 (36.9)
Primary	1605 (8.4)	1017 (5.3)	1623 (13.7)
Secondary	2824 (14.8)	3676 (19.3)	6500 (34.0)
Tertiary	859 (4.5)	2068 (10.8)	2926 (15.3)
*Religion*			
Christianity	2279 (11.9)	4556 (23.8)	6835 (35.8)
Islam	9122 (47.7)	3048 (16.0)	12,170 (63.7)
Traditional/other	68 (0.4)	28 (0.2)	95 (0.5)
*Ethnicity*			
Hausa/Fulani	7343 (38.4)	1464 (7.7)	8807 (46.1)
Igbo	403 (2.1)	1846 (9.6)	2249 (11.8)
Yoruba	550 (2.9)	1755 (9.2)	2305 (12.1)
Other	3173 (16.6)	2566 (13.4)	5740 (30.0)
*Occupation*			
Unemployed	4407 (23.1)	1733 (9.1)	6140 (32.1)
Employed	7062 (36.9)	5898 (30.9)	12,961 (67.9)
*Wealth*			
Poor	7153 (37.5)	1397 (7.3)	8550 (44.7)
Average	2274 (11.9)	1537 (8.1)	3810 (20.0)
Rich	2042 (10.7)	4698 (24.6)	6740 (35.3)
*Media exposure*			
No	8407 (44.0)	3120 (16.3)	11,527 (60.4)
Yes	3062 (16.0)	4512 (23.6)	7574 (39.6)
*Region*			
Northcentral	1315 (6.9)	1352 (7.1)	3475 (14.0)
Northeast	2594 (13.6)	881 (4.6)	3475 (18.2)
Northwest	5902 (30.9)	1115 (5.8)	7017 (36.7)
Southeast	309 (1.6)	1426 (7.5)	1735 (9.1)
South–south	758 (4.0)	823 (4.3)	1581 (8.3)
Southwest	591 (3.1)	2034 (10.7)	2625 (13.7)
*Wanted pregnancy*			
Then	10,499 (55.0)	6592 (34.5)	17,091 (89.5)
Later	694 (3.6)	703 (3.7)	1397 (7.3)
No more	275 (1.4)	336 (1.8)	612 (3.2)
*ANC visit*			
None	4459 (23.4)	229 (1.2)	4688 (24.5)
< 4 visit	2474 (12.9)	933 (4.9)	3407 (17.8)
4 + visit	4536 (23.8)	6470 (33.9)	11,006 (57.6)
*Prenatal provider*			
Unskilled	6083 (31.8)	666 (3.5)	6749 (35.3)
Skilled	5386 (28.2)	6966 (36.5)	12,352 (64.7)
*SBA use*			
No	10,698 (56.0)	502 (2.6)	11,200 (58.6)
Yes	771 (4.0)	7129 (37.4)	7900 (41.4)
*Parity*			
Primiparous	1433 (7.5)	1527 (8.0)	2960(15.5)
Multiparous	10,036 (52.5)	6105 (32.0)	16,141 (84.5)
*Delivery by CS*			
No	11,469 (60.0)	7039 (36.8)	18,508 (96.9)
Yes	0 (0.0)	592 (3.1)	593 (3.1)
*Birth type*			
Single birth	11,311 (59.2)	7436 (38.9)	18,747 (98.1)
Twin/multiple births	158 (0.8)	195 (1.1)	354 (1.9)
*Sex of child*			
Male	5811 (30.4)	3977 (20.8)	9788 (51.3)
Female	5658 (29.6)	3655 (19.1)	9313 (48.7)
*Child size*			
Small	3895 (20.4)	2789 (14.6)	6684 (35.0)
Average	5805 (30.4)	4039 (21.1)	9844 (51.5)
Large	1769 (9.2)	804 (4.2)	2573 (13.5)
*Skin-to-skin contact*			
Put to chest touching bare skin	10,544 (55.2)	5750 (30.1)	16,294 (85.3)
Put to chest not touching bare skin	216 (1.1)	318 (1.7)	534 (2.8)
Not put to chest	709 (3.7)	1564 (8.2)	2273 (11.9)
Total	11,469 (60.0)	7632 (40.0)	19,101 (100.0)

*ANC* antenatal care, *SBA* skilled birth attendant, *CS* caesarian section

### Bivariate analysis of DIBF and maternal factors relationship by place of delivery

Table 2 presents the bivariate association between DIBF status (by home and hospital delivery) and maternal factors. Age (*χ*^2^ = 37.03), residence (*χ*^2^ = 70.26), education (*χ*^2^ = 188.37), marital status (*χ*^2^ = 18.99), partner education (*χ*^2^ = 81.49), religion (*χ*^2^ = 279.00), ethnicity (*χ*^2^ = 471.60), occupation (*χ*^2^ = 2.60), wealth (*χ*^2^ = 214.77) and region (*χ*^2^ = 1100.00) are associated demographic factors with DIBF status in home delivery at *p* < 0.001 (Table 2). Obstetrics and childbirth-related factors are also significant except birth type (*χ*^2^ = 1.06, *p* = 0.304). Parity (*χ*^2^ = 6.64) and prenatal provider (*χ*^2^ = 5.11) are though significant at *p* < 0.05 (Table 2). In hospital delivery, birth type (*χ*^2^ = 1.01, *p* < 0.315) and sex of child (*χ*^2^ = 1.10, *p* < 0.295) are the only obstetric and birth factor not associated with DIBF, while media exposure (*χ*^2^ = 0.64, *p* < 0.422) is the only economic-related factor not associated with DIBF in hospital delivery. Pregnancy desire (*χ*^2^ = 4.40, *p* < 0.111) was considered as it is under the 20% cutoff point, i.e., *p* < 0.20 (Table 2).

**Table 2:** Bivariate chi-square analysis of DIBF and maternal factors by home and facility delivery

Factors	Non-institutional deliveries				Institutional deliveries			
	Timely	Delayed	*χ*^2^	*p* Value	Timely	Delayed	*χ*^2^	*p* Value
	*n* (%)	*n* (%)			*n* (%)	*n* (%)		
*Age group*			37.03	< 0.001			17.71	< 0.001
15–24	1056 (9.2)	2053 (17.9)			697 (9.1)	762 (10.0)		
25–34	2067 (18.0)	3219 (28.1)			2080 (27.3)	1897 (24.9)		
35–49	1258 (18.0)	1815 (15.8)			1129 (14.8)	1067 (14.0)		
*Place of residence*			70.26	< 0.001			8.56	0.003
Urban	1297 (11.3)	1527 (13.3)			2411 (31.6)	2151 (28.2)		
Rural	3085 (26.9)	5560 (48.8)			1495 (19.6)	1575 (20.6)		
*Education*			188.37	< 0.001			37.09	< 0.001
No formal education	2636 (23.0)	4949 (43.2)			579 (7.6)	706 (9.3)		
Primary	677 (5.9)	972 (8.5)			591 (7.7)	538 (7.1)		
Secondary	960 (8.4)	1076 (9.4)			1974 (25.9)	1778 (23.3)		
Tertiary	108 (0.9)	91 (0.8)			762 (10.0)	703 (9.2)		
*Marital status*			18.99	< 0.001			2.49	0.114
Married	4248 (37.0)	6969 (60.7)			3724 (48.8)	3587 (47.0)		
Unmarried	134 (1.2)	119 (1.0)			183 (2.4)	138 (1.8)		
*Partner education*			81.49	< 0.001			15.63	0.001
No formal education	2187 (19.1)	3994 (34.8)			419 (5.5)	451 (5.9)		
Primary	613 (5.3)	993 (8.7)			527 (6.9)	490 (6.4)		
Secondary	1222 (10.7)	1602 (14.0)			1899 (24.9)	1777 (23.3)		
Tertiary	360 (3.1)	498 (4.3)			1060 (13.9)	1007 (13.2)		
*Religion*			279.00	< 0.001			21.08	< 0.001
Christian	1129 (9.8)	1151 (10.0)			2346 (30.7)	2211 (29.0)		
Muslim	3225 (28.1)	5898 (51.4)			1544 (20.2)	1504 (19.7)		
Traditional/other	29 (0.3)	39 (0.3)			17 (0.2)	11 (0.1)		
*Ethnicity*			471.60	< 0.001			195.34	< 0.001
Hausa/Fulani	2347 (20.5)	4996 (43.6)			590 (7.7)	874 (11.5)		
Igbo	123 (1.1)	279 (2.4)			814 (10.7)	1032 (13.5)		
Yoruba	356 (3.1)	193 (1.7)			1117 (14.6)	639 (8.4)		
Other	1555 (13.6)	1618 (14.1)			1386 (18.2)	1180 (15.5)		
*Occupation*			2.60	< 0.001			29.43	< 0.001
Unemployed	1612 (14.0)	2795 (24.4)			794 (10.4)	940 (12.3)		
Employed	2770 (24.2)	4292 (37.4)			3113 (40.8)	2786 (36.5)		
*Wealth*			214.77	< 0.001			34.91	< 0.001
Poor	2368 (20.7)	4784 (41.7)			620 (8.1)	778 (10.2)		
Average	993 (8.7)	1281 (11.2)			783 (10.3)	754 (9.9)		
Rich	1020 (8.9)	1022 (8.9)			2504 (32.8)	2194 (28.7)		
*Media exposure*			6.62	0.010			0.64	0.422
No	3152 (27.5)	5254 (45.8)			1599 (20.9)	1521 (19.9)		
Yes	1229 (10.7)	1833 (16.0)			2308 (30.2)	2205 (28.9)		
*Region*			1100.00	< 0.001			398.23	< 0.001
Northcentral	802 (7.0)	513 (4.5)			836 (11.0)	516 (6.8)		
Northeast	754 (6.6)	1840 (16.0)			278 (3.6)	603 (7.9)		
Northwest	1892 (16.5)	4010 (35.0)			464 (6.1)	651 (8.5)		
Southeast	77 (0.7)	232 (2.0)			636 (8.3)	789 (10.3)		
South-south	446 (3.9)	312 (2.7)			439 (5.8)	384 (5.0)		
Southwest	410 (3.6)	181 (1.6)			1253 (16.4)	782 (10.2)		
*Wanted pregnancy*			16.00	< 0.001			4.40	0.111
Then	3956 (34.5)	6544 (57.1)			3389 (44.4)	3203 (42.0)		
Later	309 (2.7)	385 (3.4)			332 (4.4)	371 (4.9)		
No more	117 (1.0)	158 (1.4)			185 (2.4)	151 (1.9)		
*ANC visit*			42.35	< 0.001			11.40	0.003
None	1715 (14.9)	2744 (23.9)			121 (1.6)	108 (1.4)		
< 4 visit	819 (7.1)	1656 (14.4)			445 (5.8)	488 (6.4)		
4 + visit	1849 (16.1)	2687 (23.4)			3340 (43.8)	3130 (41.0)		
*Prenatal provider*			5.11	< 0.024			13.66	< 0.001
Unskilled	2242 (19.6)	3841 (33.5)			316 (4.2)	350 (4.6)		
Skilled	2139 (18.6)	3246 (28.3)			3590 (47.0)	3376 (44.2)		
*SBA use*			30.19	< 0.001			14.46	< 0.001
No	4021 (35.1)	6677 (58.2)			234 (3.1)	268 (3.5)		
Yes	360 (3.1)	411 (3.6)			3672 (48.1)	3457 (45.3)		
*Parity*			6.64	< 0.010			22.87	< 0.001
Primiparous	489 (4.3)	944 (8.2)			711 (9.3)	817 (10.7)		
Multiparous	3892 (33.9)	6144 (53.6)			3196 (41.9)	2907 (38.1)		
*Delivery by CS*							209.95	< 0.001
No	4382 (38.2)	7087 (61.8)			3787 (49.6)	3252 (42.6)		
Yes	–	–			119 (1.6)	473 (6.2)		
*Birth type*			1.06	0.304			1.01	0.315
Single birth	4326 (37.7)	6985 (60.9)			3806 (49.9)	3630 (47.6)		
Twin/Multiple births	56 (0.5)	103 (0.9)			100 (1.3)	95 (1.2)		
*Sex of child*			7.60	0.006			1.10	0.295
Male	2274 (19.8)	3536 (30.8)			1997 (26.2)	1980 (25.9)		
Female	2107 (18.4)	3550 (31.0)			1910 (25.0)	1745 (22.9)		
*Child size*			25.41	< 0.001			36.33	< 0.001
Small	1508 (13.2)	2387 (20.8)			1471 (19.3)	1319 (17.3)		
Average	2279 (19.8)	3525 (30.7)			2113 (27.7)	1926 (25.2)		
Large	594 (5.2)	1174 (10.2)			322 (4.2)	482 (6.3)		
*Skin-to-skin contact*			26.98	< 0.001			7.05	0.029
Put to chest touching bare skin	260 (2.3)	6455 (56.3)			817 (10.7)	746 (9.8)		
Put to chest no bare skin touch	33 (0.3)	183 (1.6)			120 (1.6)	198 (2.6)		
Not put to chest	4088 (35.6)	449 (3.9)			2969 (38.9)	2781 (36.5)		
Total	4382 (38.2)	7087 (61.8)			3906 (51.2)	3725 (48.8)		

*ANC* antenatal care, *SBA* skilled birth attendant, *CS* Caesarian section

### Impact of maternal characteristics of women who had home birth on DIBF

Table 3 shows adjusted and unadjusted OR and 95% CI of the association between women factors (with home births) and DIBF. Overall, women who had non-institutional delivery are 34% (adjusted effect) and 70% (unadjusted effect) more likely to delay breastfeeding initiation than those with hospital delivery {(AOR = 1.34, 95% CI 1.17–1.52); (UOR = 1.70, 95% CI 1.59–1.80)} (Table 3). Women 35–49 years are 20% less likely to DIBF compared to the 15–24 years. Odds of DIBF increase by 25% among women who had home birth and reside in rural (AOR = 1.25, 95% CI 1.10–1.38) when other factors are adjusted and by 53% when unadjusted (UOR = 1.53, 95% CI 1.40–1.67). Unadjusted odds of DIBF reduce by women and partner educational level. Odds of DIBF increase by 18% among women who had home births (AOR = 1.18, 95% CI 1.08–1.29) (Table 3). Chance of DIBF among home births reduces by 29% in middle-class  women compared to the poor (AOR = 0.71, 95% CI 0.63–0.79). Media exposure increases the odds of DIBF by 21% among women who had home birth (AOR = 1.21, 95% CI 1.09–1.34). Odds of DIBF increase by 268%, 169% and 165% in women who had home birth in northeast, northwest and southeast, respectively, but reduce by 60% in southwest compared to the northcentral. Unwanted pregnancy increases the DIBF odds by 44% among those with home births (AOR = 1.44, 95% CI 1.09–1.88) (Table 3). ANC visit less than 4 increases the odds by 52% (adjusted) and 26% (unadjusted), and DIBF chance is 9% more for female child compared to the male child of women with home birth. Skilled ANC provider and multiparity reduce DIBF chance in home births by 31% (AOR = 0.69, 95% CI 0.61–0.79) and 22% (AOR = 0.78, 95% CI 0.68–0.90), respectively. Large child size increase the odds of DIBF by 27% (adjusted) and 25% (unadjusted). Whether adjusted or not, women who do not put child to bare skin contact after home delivery are over 3 times more likely to delay breastfeeding initiation {(AOR = 3.20, 95% CI 2.11–4.85) (UOR = 3.16, 95% CI 2.11–4.71)} (Table 3).

**Table 3:** Adjusted and unadjusted odds (95% CI) of the association between DIBF and maternal characteristics by place of delivery

Factors	Non-institutional deliveries				Institutional deliveries			
	AOR	95% CI	UOR	95% CI	AOR	95% CI	UOR	95% CI
*Place of delivery*	1.34***	1.17–1.52	1.70***	1.59–1.80	0.74***	0.65–0.85	0.58***	0.55–0.63
*Age group*								
#15–24	Ref		Ref		Ref		Ref	
25–34	0.95	0.85–1.06	0.80***	0.73–0.88	1.01	0.87–1.17	0.83**	0.73–0.94
35–49	0.83**	0.73–0.94	0.74***	0.66–0.82	1.05	0.88–1.24	0.86*	0.75–0.98
*Place of residence*								
#Urban	Ref		Ref		Ref		Ref	
Rural	1.24***	1.10–1.38	1.53***	1.40–1.67	1.12	0.99–1.25	1.18***	1.07–1.29
*Education*								
#No formal education	Ref		Ref		Ref		Ref	
Primary	0.99	0.87–1.14	0.76***	0.68–0.85	1.00	0.82–1.21	0.75***	0.63–0.88
Secondary	0.95	0.82–1.11	0.59***	0.54–0.66	0.95	0.79–1.15	0.74***	0.65–0.84
Tertiary	0.81	0.57–1.14	0.44***	0.33–0.59	0.88	0.71–1.11	0.76***	0.65–0.88
*Marital status*								
#Married	Ref		Ref		Ref		Ref	
Unmarried	1.08	0.81–1.44	0.54**	0.42–0.69	0.92	0.72–1.18	0.78*	0.62–0.98
*Partner education*								
#No formal education	Ref		Ref		Ref		Ref	
Primary	1.09	0.96–1.25	0.88*	0.79–0.99	0.99	0.80–1.23	0.86	0.72–1.04
Secondary	1.27***	1.11–1.43	0.71***	0.65–0.78	1.16	0.96–1.41	0.87	0.75–1.01
Tertiary	1.24*	1.03–1.50	0.75***	0.65–0.87	1.15	0.93–1.42	0.88	0.75–1.03
*Religion*								
#Christian	Ref		Ref		Ref		Ref	
Muslim	0.86	0.74–1.02	1.79***	1.63–1.96	0.82**	0.71–0.95	1.03	0.94–1.13
Traditional/other	0.99	0.59–1.68	1.31	0.80–2.13	0.77	0.34–1.71	0.67	0.31–1.45
*Ethnicity*								
#Hausa/Fulani	Ref		Ref		Ref		Ref	
Igbo	1.29	0.85–1.94	1.06	0.85–1.32	1.13	0.85–1.49	0.85*	0.74–0.98
Yoruba	1.30	0.91–1.83	0.25***	0.21–0.30	0.72**	0.56–0.92	0.38***	0.33-.044
Other	0.63	0.55–0.73	0.48***	0.44–0.53	0.67***	0.54–0.82	0.57***	0.50–0.65
*Occupation*								
#Unemployed	Ref		Ref		Ref		Ref	
Employed	1.18***	1.08–1.29	0.89**	0.82–0.96	0.95	0.84–1.07	0.75***	0.67–0.84
*Wealth*								
#Poor	Ref		Ref		Ref		Ref	
Average	0.71***	0.63–0.79	0.64***	0.57–0.70	0.80**	0.68–0.94	0.76***	0.66–0.88
Rich	0.74***	0.63–0.86	0.49***	0.44–0.55	0.75**	0.63–0.88	0.69***	0.61–0.78
*Media exposure*								
#No	Ref		Ref		–	–	–	–
Yes	1.21***	1.09–1.34	0.89*	0.82–0.97	–	–	–	–
*Region*								
#Northcentral	Ref		Ref		Ref		Ref	
Northeast	3.68***	3.16–4.28	3.82***	3.32–4.39	3.83***	3.13–4.68	3.51***	2.93–4.20
Northwest	2.69***	2.31–3.13	3.32***	2.93–3.75	1.97***	1.58–2.45	2.27***	1.93–2.67
Southeast	2.65***	1.66–4.23	4.72***	3.56–6.25	1.29*	1.00–1.65	2.01***	1.72–2.33
South–south	0.98	0.78–1.23	1.09	0.91–1.31	1.22*	1.00–1.49	1.42***	1.19–1.69
Southwest	0.40***	0.29–0.56	0.69***	0.56–0.85	0.98	0.80–1.19	1.01	0.87–1.16
*Wanted pregnancy*								
#Then	Ref		Ref		Ref		Ref	
Later	1.17	0.98–1.39	0.75***	0.64–0.88	1.18*	1.00–1.40	1.18*	1.01–1.38
No more	1.44**	1.09–1.88	0.81	0.64–1.04	0.97	0.76–1.24	0.86	0.69–1.07
*ANC visit*								
#None	Ref		Ref		Ref		Ref	
< 4 visit	1.52***	1.31–1.76	1.26***	1.14–1.40	0.98	0.66–1.44	1.23	0.92–1.65
4 + visit	1.31***	1.14–1.51	0.90*	0.83–0.98	1.06	0.73–1.54	1.05	0.80–1.37
*Prenatal provider*								
#Unskilled	Ref		Ref		Ref		Ref	
Skilled	0.69***	0.61–0.79	0.88*	0.82–0.95	1.12	0.85–1.49	0.85*	0.72–0.99
*SBA use*								
#No	Ref		Ref		Ref		Ref	
Yes	1.13	0.94–1.34	0.68***	0.59–0.79	0.97	0.74–1.26	0.82*	0.68–0.98
*Parity*								
#Primiparous	Ref		Ref		Ref		Ref	
Multiparous	0.78**	0.68–0.90	0.81**	0.72–0.92	0.78***	0.68–0.89	0.79***	0.70–0.88
*Delivery by CS*								
#No	–	–	–	–	Ref		Ref	
Yes	–	–	–	–	5.10***	4.08–6.38	4.61***	3.75–5.67
*Sex of child*								
#Male	Ref		Ref		**–**	**–**	**–**	**–**
Female	1.09*	1.00–1.17	1.08*	1.00–1.17	**–**	**–**	**–**	**–**
*Child size*								
#Small	Ref		Ref		Ref		Ref	
Average	1.07	0.98–1.17	0.97	0.89–1.06	1.05	0.94–1.17	1.02	0.92–1.12
Large	1.27***	1.12–1.44	1.25***	1.10–1.40	1.64***	1.38–1.94	1.67***	1.42–1.95
*Skin-to-skin contact*								
#Put to chest touching bare skin	Ref		Ref		Ref		Ref	
Put to chest no bare skin touch	3.20***	2.11–4.85	3.16***	2.11–4.71	1.22	0.91–1.62	1.82***	1.41–2.33
Not put to chest	0.95	0.80–1.13	0.91	0.77–1.07	1.17*	1.03–1.33	1.03	0.91–1.15

*ANC* antenatal care, *SBA* skilled birth attendant, *CS* caesarian section

*Significant at *p* < 0.05, **significant at *p* < 0.01, ***significant at *p* < 0.001, #Reference category

### Impact of maternal characteristics of women who had hospital birth on DIBF

Hospital delivery effect on DIBF is shown in Table 3. Overall, odds of DIBF reduce by 26% (adjusted effect) and 42% (unadjusted effect) among women who had hospital delivery compared to home births {(AOR = 0.74, 95% CI 0.65–0.85) (AOR = 0.58, 95% CI 0.55–0.63)} (Table 3). Odds of DIBF decrease by 20% (AOR = 0.80, 95% CI 0.68–0.94) and 25% (AOR = 0.75, 95% CI 0.63–0.88) among average and rich women with hospital births compared to the poor. Muslim women who had hospital birth are 1.22 times less likely to delay than Christians (AOR = 0.82, 95% CI 0.71–0.95). Yoruba and other ethnic are 39% and 49% times less likely to delay. Odds of DIBF for institutional birth increase by 283% in northeast, 97% in northwest, 29% in southeast and 22% in south–south compared to the northcentral. Rural women increase the odds by 18% when unadjusted with other factors (UOR = 1.18, 95% CI 1.07–1.29) (Table 3). Women who wanted pregnancy later and had hospital delivery are 18% times more likely to delay {(AOR = 1.18, 95% CI 1.00–1.40) (UOR = 1.18, 95% CI 1.01–1.38)}. Those who had caesarian births are about 5 times more likely to delay than those who had vaginal births {(AOR = 5.10, 95% CI 4.08–6.38) (UOR = 4.61, 95% CI 3.75–5.67)}. Large child size after hospital birth increases DIBF chance by 64% and 67% when other variables are adjusted and unadjusted, respectively. The odds of DIBF increase by 82% when child is put to chest but not touching bare skin (UOR = 1.82, 95% CI 1.41–2.33) and increase by 17% when child is not put to chest after hospital birth (AOR = 1.17, 95% CI 1.03–1.33) (Table 3).

### Decomposing effect of non-institutional delivery on DIBF

Table 4 presents the decomposition of non-hospital delivery effect on DIBF. Women aged 35–49 minimally reduce the home-hospital delivery gap and contributed 13.8%. Home-facility birth gap can be reduced by 13.7% if urban distribution is similar to rural, while secondary and tertiary educated partners increase the gap by 10.1% and 7.9%, respectively (Table 4). Yoruba and employed women significantly reduce the DIBF effect by 23.7% compared to the Hausa and unemployed women, respectively. Rich women reduce the gap by 23.9% compared to the poor (Table 4). Women home–hospital delivery variation reduces by 26.3%, 66.5% and 35.4% for DIBF in northeast, northwest and southwest, respectively, and increases by 28.4% in southeast. Southeast and southwest contribute to 25.7% and − 40.4% of the non-institutional delivery coefficient effect on DIBF, respectively (Table 4). Undesired pregnancy increases the home–hospital delivery gap by 1.3% and contributes to 2.8% of DIBF effect. ANC visit < 4 reduces the gap by 7.1% with 8.8% contribution to DIBF effect, while 4 or more ANC visit raises the gap by 22.5%. Using skilled provider at ANC reduces the home–hospital difference by 29.3% and significantly contributes to 66.3% DIBF effect (Table 4). Multiparous women reduce the gap by 3.3%, and female child reduces the gap and contributes to 13.9% of DIBF effect. Large child size raises the gap by 2.1% with 4.3% DIBF effect. Compared to children placed on chest and touching bare skin, placing child on chest not touching bare skin raises the gap by 7.9% and significantly increase DIBF effect by 60.6%. Overall, 69% (*p* < 0.001) of the DIBF effect were due to characteristics/endowment component, while 31% (*p* < 0.005) of the effect were due to the coefficient’s components (Table 4).

**Table 4:** Decomposition analysis of non-institutional delivery effect on DIBF

Factors	Effect due to characteristics(C)			Effect due to coefficients(E)		
	Coefficients	*p* Value	Percent	Coefficients	*p* Value	Percent
*Age group*						
#15–24	Ref			Ref		
25–34	0.00070	0.375	0.54	− 0.00712	0.488	− 5.48
35–49	0.00085	0.004	0.66	− 0.01797	0.006	− 13.84
*Place of residence*						
#Urban	Ref			Ref		
Rural	0.01781	0.000	13.72	0.00935	0.183	7.20
*Education*						
#No formal education	Ref			Ref		
Primary	0.00001	0.951	0.01	0.00162	0.667	1.25
Secondary	0.00344	0.548	2.65	0.00388	0.760	2.99
Tertiary	0.00865	0.229	6.66	− 0.00620	0.472	− 4.78
*Marital status*						
#Married	Ref			Ref		
Unmarried	− 0.00038	0.580	− 0.29	0.00173	0.319	1.33
*Partner education*						
#No formal education	Ref			Ref		
Primary	0.00014	0.175	0.11	0.00208	0.568	1.61
Secondary	− 0.01310	0.000	− 10.09	0.01071	0.375	8.25
Tertiary	− 0.01009	0.021	− 7.78	0.00367	0.662	2.83
*Religion*						
#Christian	Ref			Ref		
Muslim	− 0.01322	0.082	− 10.82	0.00745	0.428	5.74
Traditional/other	− 0.00001	0.999	− 0.01	0.00023	0.549	0.18
*Ethnicity*						
#Hausa/Fulani	Ref			Ref		
Igbo	− 0.01233	0.228	− 9.50	0.00221	0.867	1.70
Yoruba	− 0.01117	0.137	− 8.60	0.03078	0.005	23.71
Other	0.00640	0.000	4.93	− 0.00485	0.591	− 3.73
*Occupation*						
#Unemployed	Ref			Ref		
Employed	− 0.00624	0.000	− 4.81	0.03078	0.005	23.71
*Wealth*						
#Poor	Ref			Ref		
Average	0.00024	0.000	0.19	− 0.00462	0.287	− 3.56
Rich	0.03105	0.000	23.92	0.00062	0.967	0.48
*Media exposure*						
#No	Ref			Ref		
Yes	− 0.01489	0.000	− 11.47	0.00228	0.811	1.75
*Region*						
#Northcentral	Ref			Ref		
Northeast	0.03413	0.000	26.29	− 0.00078	0.806	0.60
Northwest	0.08628	0.000	66.46	0.01003	0.019	7.73
Southeast	− 0.03690	0.000	− 28.42	0.03335	0.002	25.69
South–south	0.00015	0.895	0.11	− 0.00467	0.183	− 3.60
Southwest	0.04598	0.000	35.42	− 0.05244	0.000	− 40.39
*Wanted pregnancy*						
#Then	Ref			Ref		
Later	− 0.00116	0.081	− 0.90	− 0.00074	0.762	− 0.57
No more	− 0.00172	0.009	− 1.32	0.00366	0.036	2.82
*ANC visit*						
#None	Ref			Ref		
< 4 visit	0.00924	0.000	7.12	0.01149	0.038	8.85
4 + visit	− 0.02917	0.000	− 22.47	0.03566	0.335	27.47
*Prenatal provider*						
#Unskilled	Ref			Ref		
Skilled	0.03798	0.000	29.25	− 0.08606	0.005	− 66.29
*SBA use*						
#No	Ref			Ref		
Yes	− 0.02472	0.186	− 19.04	0.01539	0.633	11.86
*Parity*						
#Primiparous	Ref			Ref		
Multiparous	− 0.00430	0.001	− 3.31	0.00468	0.784	3.61
*Sex of child*						
#Male	Ref			Ref		
Female	0.00028	0.042	0.22	0.01804	0.006	13.90
*Child size*						
#Small	Ref			Ref		
Average	− 0.00038	0.125	− 0.29	0.00514	0.516	3.96
Large	0.00278	0.000	2.14	− 0.00563	0.021	− 4.34
*Skin-to-skin contact*						
#Put to chest touching bare skin	Ref			Ref		
Put to chest no bare skin touch	− 0.01030	0.003	− 7.94	− 0.07871	0.000	− 60.63
Not put to chest	− 0.00628	0.000	− 4.84	0.00310	0.168	2.39
Constant				0.05416	0.391	41.72
E/C	0.08976	0.000	69.14	0.04006	0.18	30.86
R	0.12982	0.000				

^#^ Reference category, *E* endowment component, *C* coefficient component, *R* residual, *ANC* antenatal care, *SBA* skilled birth attendant

### Subnational effect on delayed initiation of breastfeeding

Table 5 shows the state-level prevalence and impact on DIBF in reference to the highest women population in Kano. Odds of DIBF are approximately 5 times more likely in Jigawa (OR = 5.43, 95% CI 4.24–6.94) and Taraba (OR = 4.92, 95% CI 3.74–6.48) than Kano. Women in Bauchi (OR = 4.25, 95% CI 3.37–5.35) and Sokoto (OR = 3.93, 95% CI 3.05–5.06) are approximately 4 times as likely as Kano Women to delay breastfeeding initiation. Odds of DIBF are also positive and significant in Adamawa (OR = 2.63, 95% CI 2.07–3.34), Gombe (OR = 2.58, 95% CI 2.08–3.20), Nasarawa (OR = 2.01, 95% CI 1.58–2.56) and Kebbi (OR = 1.68, 95% CI 1.38–2.05). On the other hand, odds of DIBF are about 50 times less likely in Bayelsa compared to Kano (OR = 0.02, 95% CI 0.01–0.04). Oyo (OR = 0.17, 95% CI 0.13–0.23), Ogun (OR = 0.18, 95% CI 0.14–0.25) and Kogi (OR = 0.18, 95% CI 0.13–0.24) women are about 6 times less likely to delay breastfeeding initiation than Kano Women. Odds of DIBF reduced by 78% in Niger, 65% in cross-rivers, 64% in Osun and Edo, 63% in Benue and 62% in Abuja when compared with Kano.

**Table 5:** State-level analysis of delayed initiation of breastfeeding

States	Initiation of Breastfeeding			OR	95% CI	*p* Value
	Delayed *n* (%)	Timely *n* (%)	All *n* (%)			
#Kano	938 (4.91)	640 (3.35)	1578 (8.26)	Ref		
Sokoto	469 (2.45)	71 (0.37)	540 (2.83)	3.93	3.05–5.06	< 0.001
Zamfara	372 (1.95)	387 (2.03)	759 (3.98)	0.62	0.51–0.75	< 0.001
Katsina	804 (4.21)	482 (2.5)	1286 (6.73)	1.04	0.86–1.25	0.661
Jigawa	767 (4.02)	88 (0.46)	855 (4.48)	5.43	4.24–6.94	< 0.001
Yobe	324 (1.70)	351 (1.84)	675 (3.53)	0.61	0.50–0.74	< 0.001
Borno	333 (1.74)	350 (1.84)	683 (3.58)	0.62	0.51–0.75	< 0.001
Adamawa	366 (1.92)	88 (0.46)	454 (2.38)	2.63	2.07–3.34	< 0.001
Gombe	320 (1.68)	73 (0.38)	393 (2.06)	2.58	2.08–3.20	< 0.001
Bauchi	725 (3.80)	124 (0.65)	849 (4.45)	4.25	3.37–5.35	< 0.001
Kaduna	825 (4.32)	495 (2.59)	1320 (6.91)	1.19	0.99–1.44	0.058
Kebbi	485 (2.54)	193 (1.01)	678 (3.55)	1.68	1.38–2.05	< 0.001
Niger	187 (0.98)	574 (3.0)	761 (3.99)	0.22	0.17–0.27	< 0.001
FCT Abuja	50 (0.26)	76 (0.40)	126 (0.66)	0.38	0.30–0.48	< 0.001
Nasarawa	217 (1.14)	74 (0.39)	291 (1.53)	2.01	1.58–2.56	< 0.001
Plateau	176 (0.92)	155 (0.81)	331 (1.73)	0.74	0.59–0.93	0.011
Taraba	374 (1.96)	45 (0.24)	419 (2.20)	4.92	3.74–6.48	< 0.001
Benue	206 (1.08)	348 (1.82)	554 (2.90)	0.37	0.30–0.46	< 0.001
Kogi	56 (0.29)	210 (1.10)	266 (1.40)	0.18	0.13–0.24	< 0.001
Kwara	136 (0.71)	200 (1.05)	336 (1.76)	0.40	0.32–0.51	< 0.001
Oyo	135 (0.71)	479 (2.51)	614 (3.22)	0.17	0.13–0.23	< 0.001
Osun	138 (0.72)	244 (1.28)	382 (2.00)	0.36	0.28–0.46	< 0.001
Ekiti	88 (0.46)	107 (0.56)	195 (1.02)	0.55	0.42–0.71	< 0.001
Ondo	136 (0.71)	111 (0.58)	247 (1.29)	0.74	0.57–0.95	0.017
Edo	80 (0.42)	127 (0.67)	207 (1.08)	0.36	0.27–0.48	< 0.001
Anambra	334 (1.75)	212 (1.11)	546 (2.86)	1.07	0.85–1.33	0.570
Enugu	137 (0.72)	124 (0.65)	261 (1.37)	0.70	0.54–0.91	0.007
Ebonyi	259 (1.36)	152 (0.80)	411 (2.16)	1.06	0.85–1.31	0.565
Cross River	58 (0.30)	103 (0.54)	161 (0.85)	0.35	0.26–0.47	< 0.001
Akwa Ibom	127 (0.66)	157 (0.82)	184 (1.49)	0.51	0.39–0.66	< 0.001
Abia	102 (0.53)	89 (0.47)	191 (1.00)	0.82	0.63–1.07	0.153
Imo	188 (0.99)	135 (0.71)	324 (1.70)	0.76	0.60–0.97	0.029
Rivers	249 (1.31)	260 (1.36)	509 (2.67)	0.59	0.46–0.75	< 0.001
Bayelsa	2 (0.01)	98 (0.51)	100 (0.52)	0.02	0.01–0.04	< 0.001
Delta	180 (0.94)	138 (0.72)	318 (1.67)	0.83	0.64–1.09	0.196
Lagos	388 (2.03)	440 (2.31)	828 (4.34)	0.57	0.45–0.71	< 0.001
Ogun	77 (0.41)	281 (1.47)	358 (1.88)	0.18	0.14–0.25	< 0.001
Total	10,813 (56.61)	8288 (43.39)	19,101 (100)			

^#^Reference category

## Discussion

The role of place of delivery on delayed initiation of breastfeeding, i.e., beyond the first hour of newborn life recommended by WHO and UNICEF, was investigated. This was evaluated via decomposition analysis of non-institutional delivery effect on DIBF, and the prospect of DIBF across subnational prevalence was also determined. The study findings will be helpful in providing evidence to support strategic development of intervention for optimal breastfeeding practice.

A 56.6% (43.4% initiated breastfeeding early) prevalence of delayed breastfeeding initiation was found in Nigeria. This was lower among women who had hospital delivery compared to those that had home birth, and about two-third of the DIBF prevalence was among women who delivered at home, while the one-third was among those who had hospital birth. In total, the prevalence of home and hospital delivery were 60% and 40%, respectively. These findings agree with studies assessing prevalence and determinants of breastfeeding and facility-based delivery in Nigeria [14, 18, 33, 34].

Similar demographic factors were selected in the bivariate association with DIBF among mothers who had institutional and non-institutional deliveries. Birth type as an obstetric factor is surprisingly not associated with the DIBF among women who had both hospital and home delivery, while media exposure as an economic-related factors is not associated with DIBF in hospital delivery only. Comparable factors were identified in studies assessing TIBF and DIBF that applied chi-square statistics to select variables for inclusion in the multivariable analysis [42, 43].

The pool impact of place of delivery on DIBF was significant as women who had non-institutional delivery have a 34% chance of delaying breastfeeding initiation. Correspondingly, the risk of delaying breastfeeding initiation reduces by 26% among women who had health facility-based delivery which is in congruent with studies on DIBF [29]. Caesarian section delivery is associated with DIBF only among women with institutional delivery as those that delivered at home are expected to have the normal vaginal birth since the traditional birth attendant/person expectedly lack the require expertise to perform CS birth. Hence, those who delivered via CS are about 5 times more likely to delay breastfeeding initiation and this finding is in concurrence with factors identified in studies investigating obstetrics effect on DIBF [11, 29, 41]. Whether women had home or facility-based delivery, undesired pregnancy, large child size at birth and partial/no skin-to-skin contact positively influence the delayed of breastfeeding initiation, while negative influence on DIBF was observed among the multiparas, women in wealth quintiles, in high reproductive age (35–49 years) and utilization of skilled providers during prenatal care. This clearly highlights the substantive impact of maternal experience as women in this class are likely to be nursing at least the second child as well as the effect of skilled health providers and the financial power to sought and utilize reproductive health services required to improve mother and child health [44–46].

Furthermore, rural women who had home birth have 24% likelihood of delaying breastfeeding initiation than their urban counterparts. Studies in SSA also reported analogous findings due to difference in urbanization as women in urban have more access to institutional infrastructure [19, 47]. Partner education media exposure and ANC visit have a reverse effect on the women who had home birth as having partner with higher education, being exposed to media and 4 or more ANC increases the chance of delaying breastfeeding initiation when compared with having partner without formal education, not exposed to media and zero ANC visit, respectively. Studies have provide evidence to support the educational effect and the ANC effect which is attributable to the delay in initiation [48–50]. Women in the northeast, northwest and southeast are more likely to delay initiation of breastfeeding irrespective of the type of place of delivery. The chance of late breastfeeding initiation reduces by 28% and 33% among the Yoruba and Other ethnic women compared to the Hausa/Fulani. South–south women who had institutional birth are more likely to delay initiation of breastfeeding, while women in the southwest who had home birth are less likely to delay breastfeeding initiation. Giving birth to female child after home birth increases the chance of delaying breastfeeding initiation by 9% and thus highlights the contribution to DIBF effect on neonatal morbidity with lower risk of infant survival [4, 7].

The decomposition of factors revealed that the difference in home-facility birth was reduced by 29.3% among women who utilized skilled provider with significant 66.3% contribution to the raise in the DIBF effect. Implying that utilization of skilled provider will reduce the institutional and non-institutional gap by about one-third and help alleviate two-third of adverse DIBF effect as corroborated by the pre- and post-breastfeeding training impact on EIBF in Sudan [51]. Home-facility delivery gap will significantly reduce if women who practiced partial/no SSC have the distribution similar to their counterparts with full SSC and therefore contributed to 60.6% DIBF effect, thus highlighting the importance of full SSC practice in minimizing the gap in home-facility birth and isolating three-fifth of the DIBF effect as reported in recent studies [26–28]. Southwest women reduce the gap, while southeast women increase the gap. Hence, home–hospital birth variation and the negative DIBF effect can be reduced by the respective 35.4% and 40.4% if breastfeeding practice in the southwest is upheld in the northcentral. Also, increase in access to quality healthcare in the rural will translate to reduction in home–hospital delivery gap by 13.7% if the distribution of urban women is observed in rural. Women 35–49 years positively contribute (13.8%) to the DIBF effect, and the difference in home and hospital birth for DIBF due to partner education was significant. Yoruba and unemployed women importantly contribute to the 23.7% reduction in DIBF effect when compared with Hausa/Fulani and unemployed women, respectively. Overall, about 69% of the decomposed component of the delayed breastfeeding effect were due to the characteristics/endowment effect, while 31% were due to the coefficient effect.

### Regional-level implications

Based on subnational analysis, DIBF was most prevalent in Kano and least prevalent in Bayelsa. The likelihood of delaying breastfeeding initiation was approximately 5 times more likely in Jigawa and Taraba than Kano. The chance of delaying breastfeeding initiation is about 4 times as likely in Sokoto and Bauchi states as Kano. Also, the odd of DIBF is positive and significant in Adamawa, Gombe, Nasarawa and Kebbi states. However, odds of delaying initiation of breastfeeding are 50 times less likely in Bayelsa and 6 times less likely in Oyo and Ogun. This corresponds to findings from sub-country level analysis in Nigeria [28]. DIBF was also less likely in Niger, Cross-rivers, Osun, Edo, Benue, Abuja among other states when compared to Kano. This is an indication for the need to improve breastfeeding practice in the north based on lesson learnt in the south.

### Study strengths and limitations

The study might have been affected by recall bias majorly associated with cross-sectional studies. This was minimized by the analysis of a weighted sample of respondents with children less than 5 years. The study could not ascertain that non-facility delivery is a cause of delayed initiation of breastfeeding as other criteria for causality were not assessed. Hence, interpretations of findings should be limited to associations. The author was also limited to the choice of study variables as collected in the operationalized DHS. Study strengths can, however, be observed from the applicability of weighted survey data which is a representative of the target population. Therefore, improved the reliability of the study estimates and the generalizability of the study findings herein. The application of decomposition analysis technique to determine percentage contribution per effect size is also a strength. This is the first study that decompose the effect of prevailing non-institutional delivery on breastfeeding and therefore provides evidence-based strategy for implementation of intervention for the group (fraction to treat) to improve breastfeeding practice.

## Conclusions

More than half of women delayed initiation of breastfeeding for newborns in Nigeria and about two-third and one-third of the prevalent was found in home and facility delivery, respectively. The likelihood of delaying breastfeeding initiation increases by 34% in home birth and decreases by 26% in facility delivery. Undesired pregnancy, rural residency, practice of no/partial skin-to-skin contact and large child size at birth are significantly associated with delay initiation of breastfeeding in home and facility-based delivery. Utilization of skilled provider at ANC, parity and wealth significantly reduce the chance of delaying breastfeeding initiation in home birth while the chance of breastfeeding initiation delay is 5 times more likely among women who had caesarian birth compared to virginal birth in a health facility. ANC provider, region and wealth contributed most to the characteristics effect, while partial skin-to-skin contact and skilled ANC provider contributed most to the adverse effect due to home-facility gap. Breastfeeding delay is most prevalent in Kano, more likely in Bauchi and Sokoto and less likely in Bayelsa, Oyo, Ogun among other subnational.

## Recommendations

The study findings highlighted the need for governmental and non-governmental organizations to intensify on sensitization and follow-up maternity programs action that promote facility-based delivery if Nigeria is to come close to achieving the 2030 sustainable development goal for maternal and childbirth indicators. Gap in home-facility delivery and the adverse effect of delayed breastfeeding initiation can be zeroized by increasing access to skilled provider at antenatal and strengthening full uptake of skin-to-skin contact after childbirth while discouraging home delivery particularly in the rural communities. The north should emulate facility-based delivery and optimal breastfeeding practice in the south and learn from what does not work in the region.

### Supplementary Information

**Additional file 1.** STROBE Statement—Checklist of items that should be included in reports of cross-sectional studies.

## Data Availability

The de-identified data are available in the public domain. Dataset used (generated and/or analyzed) in this current study is available on reasonable request from the corresponding author and can be accessed at the open repository of the DHS program www.dhsprogram.com.

## Abbreviations

AOR: Adjusted odds ratio
CI: Confidence interval
DIBF: Delayed initiation of breastfeeding
IMR: Infant mortality rate
IYCF: Infant and young child feeding
NDHS: Nigerian Demographic and Health Survey
NMR: Neonatal mortality rate
NPHC: National Population and Housing Census
SDG: Sustainable development goal
SSA: Sub-Saharan Africa
SSC: Skin-to-skin contact
TIBF: Timely initiation of breastfeeding
U5MR: Under-5 mortality rate
UNICEF: United Nations Children’s Fund
UOR: Unadjusted odds ratio
WHO: World Health Organization

## References

[1] WHO_UNICEF_Infant and young child feeding.

[2] Hailemariam TW, Adeba E, Sufa A (2015). Predictors of early breastfeeding initiation among mothers of children under 24 months of age in rural part of West Ethiopia Global health. BMC Public Health.

[3] Babatunde Yahya W, Adebayo SB (2013). Modelling the trend and determinants of breastfeeding initiation in Nigeria. Child Dev Res.

[4] Smith ER, Hurt L, Chowdhury R, Sinha B, Fawzi W, Edmond KM (2017). Delayed breastfeeding initiation and infant survival: a systematic review and meta-analysis. PLoS ONE.

[5] Raihana S, Dibley MJ, Rahman MM, Tahsina T, Siddique MAB, Rahman QS (2019). Early initiation of breastfeeding and severe illness in the early newborn period: an observational study in rural Bangladesh. PLoS Med.

[6] Örün E, Yalçin SS, Madendaǧ Y, Üstünyurt-Eras Z, Kutluk Ş, Yurdakök K (2010). Factors associated with breastfeeding initiation time in a Baby-Friendly Hospital. Turk J Pediatr.

[7] Oyedele OK, Fagbamigbe AF, Ayeni O. Modelling time-to-discontinuation of exclusive breastfeeding: Analysis of infants and under-2 survival in Nigeria. Etude la Popul Afr. 2020;34(1).

[8] Nkoka O, Ntenda PAM, Kanje V, Milanzi EB, Arora A (2019). Determinants of timely initiation of breast milk and exclusive breastfeeding in Malawi: a population-based cross-sectional study. Int Breastfeed J.

[9] Issaka AI, Agho KE, Renzaho AMN (2017). Prevalence of key breastfeeding indicators in 29 sub-Saharan African countries: a meta-analysis of demographic and health surveys (2010–2015). BMJ Open.

[10] Birhanu A, Id T, Tesema GA (2021). Timely initiation of breastfeeding and associated factors among mothers having children less than two years of age in sub- Saharan Africa: a multilevel analysis using recent. Demogr Health Surv Data.

[11] Mukunya D, Tumwine JK, Nankabirwa V, Ndeezi G, Odongo I, Tumuhamye J (2017). Factors associated with delayed initiation of breastfeeding: a survey in Northern Uganda. Glob Health Action.

[12] Dubik SD, Amegah KE (2021). Prevalence and determinants of early initiation of breastfeeding (EIBF) and prelacteal feeding in Northern Ghana: a cross-sectional survey. PLoS ONE.

[13] National Population Commission(NPC)[Nigeria], ICF International. Nigeria Demograhic Health Survey, 2013. Abuja; 2014.

[14] National Population Commission(NPC)[Nigeria], ICF International. Nigeria Demographic and Health Survey 2018. Abuja, Nigeria, And Rockville, Maryland, USA; 2019.

[15] United Nations. Sustainable Development Goals (SDG). Washington, DC; 2015.

[16] Phukan D, Ranjan M, Dwivedi LK (2018). Impact of timing of breastfeeding initiation on neonatal mortality in India. Int Breastfeed J.

[17] Takahashi K, Ganchimeg T, Ota E, Vogel JP, Souza JP, Laopaiboon M (2017). Prevalence of early initiation of breastfeeding and determinants of delayed initiation of breastfeeding: secondary analysis of the WHO Global Survey. Sci Rep.

[18] Berde AS, Yalcin SS (2016). Determinants of early initiation of breastfeeding in Nigeria: a population-based study using the 2013 demograhic and health survey data. BMC Pregnancy Childbirth.

[19] Mekonen L, Seifu W, Shiferaw Z (2018). Timely initiation of breastfeeding and associated factors among mothers of infants under 12 months in South Gondar zone, Amhara regional state, Ethiopia; 2013. Int Breastfeed J.

[20] Alebel A, Dejenu G, Mullu G, Abebe N, Gualu T, Eshetie S (2017). Timely initiation of breastfeeding and its association with birth place in Ethiopia: a systematic review and meta-analysis. Int Breastfeed J.

[21] Ndirangu MN, Gatimu SM, Mwinyi HM, Kibiwott DC (2018). Trends and factors associated with early initiation of breastfeeding in Namibia: analysis of the Demographic and Health Surveys 2000–2013. BMC Pregnancy Childbirth.

[22] Woldeamanuel BT (2020). Trends and factors associated to early initiation of breastfeeding, exclusive breastfeeding and duration of breastfeeding in Ethiopia: evidence from the Ethiopia Demographic and Health Survey 2016. Int Breastfeed J.

[23] John JR, Mistry SK, Kebede G, Manohar N, Arora A (2019). Determinants of early initiation of breastfeeding in Ethiopia: a population-based study using the 2016 demographic and health survey data. BMC Pregnancy Childbirth.

[24] Ali F, Mgongo M, Mamseri R, George JM, Mboya IB, Msuya SE (2020). Prevalence of and factors associated with early initiation of breastfeeding among women with children aged < 24 months in Kilimanjaro region, northern Tanzania: a community-based cross-sectional study. Int Breastfeed J.

[25] Oakley L, Benova L, Macleod D, Lynch CA, Campbell OMR (2018). Early breastfeeding practices: descriptive analysis of recent Demographic and Health Surveys. Matern Child Nutr.

[26] Obioha CU, Martin MP, Obioha OA, Padron-Monedero A (2021). Association between skin-to-skin contact post-birth and breastfeeding behaviour: a cross-sectional study of Nigerian women using the 2018 Demographic Health Survey. J Glob Heal Rep.

[27] Singh K, Khan SM, Carvajal-Aguirre L, Brodish P, Amouzou A, Moran A (2017). The importance of skin-to-skin contact for early initiation of breastfeeding in Nigeria and Bangladesh. J Glob Health.

[28] Ekholuenetale M, Barrow A, Arora A (2022). Skin-to-skin contact and breastfeeding practices in Nigeria: a study of socioeconomic inequalities. Int Breastfeed J.

[29] Raihana S, Alam A, Chad N, Huda TM, Dibley MJ (2021). Delayed initiation of breastfeeding and role of mode and place of childbirth: evidence from health surveys in 58 low-and middle-income countries (2012–2017). Int J Environ Res Public Health.

[30] Ayesha U, Mamun ASMA, Sayem MA, Hossain MG (2021). Factors associated with duration of breastfeeding in Bangladesh: evidence from Bangladesh demographic and health survey 2014. BMC Public Health.

[31] Birhan TY, Birhan NA, Alene M (2021). Pooled prevalence and determinants of prelacteal feeding practice in eastern Africa evidence from demographic and health survey data: a multilevel study. Risk Manag Healthc Policy.

[32] Ogunkunle TO, Gabriel TY, Bello SO, Abdullahi Y, Bulus J, Ozhe SI (2021). Facility-based newborn deaths at a referral tertiary hospital in North-Central Nigeria during the sustainable development goal era: a retrospective cohort analysis. J Trop Pediatr.

[33] Adewuyi EO, Zhao Y, Auta A, Lamichhane R (2017). Prevalence and factors associated with non-utilization of healthcare facility for childbirth in rural and urban Nigeria: analysis of a national population-based survey. Scand J Public Health.

[34] Adewuyi EO, Khanal V, Zhao Y, David L, Bamidele OD, Auta A (2019). Home childbirth among young mothers aged 15–24 years in Nigeria: a national population-based cross-sectional study. BMJ Open.

[35] Bolarinwa OA, Fortune E, Aboagye RG, Seidu AA, Olagunju OS, Nwagbara UI (2021). Health facility delivery among women of reproductive age in Nigeria: does age at first birth matter?. PLoS ONE.

[36] Fagbamigbe AF, Oyedele OK (2022). Multivariate decomposition of trends, inequalities and predictors of skilled birth attendants utilisation in Nigeria (1990–2018): a cross-sectional analysis of change drivers. BMJ Open.

[37] Fagbamigbe AF, Hurricane-Ike EO, Yusuf OB, Idemudia ES (2017). Trends and drivers of skilled birth attendant use in Nigeria (1990–2013): policy implications for child and maternal health. Int J Womens Health.

[38] Fagbamigbe AF, Bello S, Salawu MM, Afolabi RF, Gbadebo BM, Adebowale AS (2021). Trend and decomposition analysis of risk factors of childbirths with no one present in Nigeria, 1990–2018. BMJ Open.

[39] Nation U. World population projections. Uma ética para quantos? 2015;XXXIII(2):81–7.

[40] Powers DA, Yoshioka H, Yun M-S (2011). Mvdcmp: multivariate decomposition for nonlinear response models. Stata J Promot Commun Stat Stata.

[41] Oyedele OK (2023). Effect of caesarian section delivery on breastfeeding initiation in Nigeria: logit-based decomposition and subnational analysis of cross-sectional survey. BMJ Open.

[42] Horii N, Allman J, Martin-Prével Y, Waltisperger D (2017). Determinants of early initiation of breastfeeding in rural Niger: cross-sectional study of community based child healthcare promotion. Int Breastfeed J.

[43] Taha Z, Hassan AA, Wikkeling-Scott L, Papandreou D (2021). Factors associated with delayed initiation and cessation of breastfeeding among working mothers in Abu Dhabi, the United Arab Emirates. Int J Womens Health.

[44] Manyeh AK, Amu A, Akpakli DE, Williams JE, Gyapong M (2020). Estimating the rate and determinants of exclusive breastfeeding practices among rural mothers in Southern Ghana. Int Breastfeed J.

[45] Babalola S, Fatusi A (2009). Determinants of use of maternal health services in Nigeria—looking beyond individual and household factors. BMC Pregnancy Childbirth.

[46] Oyedele OK, Fagbamigbe AF, Akinyemi OJ, Adebowale AS (2023). Coverage-level and predictors of maternity continuum of care in Nigeria: implications for maternal, newborn and child health programming. BMC Pregnancy Childbirth.

[47] Adewuyi EO, Zhao Y, Khanal V, Auta A, Bulndi LB (2017). Rural-urban differences on the rates and factors associated with early initiation of breastfeeding in Nigeria: further analysis of the Nigeria demographic and health survey, 2013. Int Breastfeed J.

[48] Acharya P, Khanal V (2015). The effect of mother’s educational status on early initiation of breastfeeding: further analysis of three consecutive Nepal Demographic and Health Surveys Global health. BMC Public Health.

[49] Wako WG, Wayessa Z, Fikrie A (2022). Effects of maternal education on early initiation and exclusive breastfeeding practices in sub-Saharan Africa: a secondary analysis of Demographic and Health Surveys from 2015 to 2019. BMJ Open.

[50] Aliyu AA, Dahiru T (2017). Predictors of delayed antenatal care (ANC) visits in Nigeria: secondary analysis of 2013 Nigeria Demographic and Health Survey (NDHS). Pan Afr Med J.

[51] Tongun JB, Tumwine JK, Ndeezi G, Sebit MB, Mukunya D, Nankunda J (2019). The effect of health worker training on early initiation of breastfeeding in south Sudan: a hospital-based before and after study. Int J Environ Res Public Health.

